# IL-21/IL-21R Regulates the Neutrophil-Mediated Pathologic Immune Response during Chlamydial Respiratory Infection

**DOI:** 10.1155/2022/4322092

**Published:** 2022-06-01

**Authors:** Jiajia Zeng, Yueyue Xu, Lu Tan, Xiaoyu Zha, Shuaini Yang, Hong Zhang, Yuqing Tuo, Ruoyuan Sun, Wenhao Niu, Gaoju Pang, Lida Sun, Hong Bai

**Affiliations:** Department of Immunology, Key Laboratory of Immune Microenvironment and Disease of the Educational Ministry of China, Tianjin Key Laboratory of Cellular and Molecular Immunology, School of Basic Medical Sciences, Tianjin Medical University, Tianjin, China

## Abstract

IL-21/IL-21R was documented to participate in the regulation of multiple infection and inflammation. During *Chlamydia muridarum* (*C. muridarum*) respiratory infection, our previous study had revealed that the absence of this signal induced enhanced resistance to infection with higher protective Th1/Th17 immune responses. Here, we use the murine model of *C. muridarum* respiratory infection and IL-21R deficient mice to further identify a novel role of IL-21/IL-21R in neutrophilic inflammation. Resistant IL-21R^−/−^ mice showed impaired neutrophil recruitment to the site of infection. In the absence of IL-21/IL-21R, pulmonary neutrophils also exhibited reduced activation status, including lower CD64 expression, MPO activity, and neutrophil-produced protein production. These results correlated well with the decrease of neutrophil-related chemokines (KC and MIP-2), inflammatory cytokines (IL-6, IL-1*β*, and TNF-*α*), and TLR/MyD88 pathway mediators (TLR2, TLR4, and MyD88) in infected lungs of IL-21R^−/−^ mice than normal mice. Complementarily, decreased pulmonary neutrophil infiltration, activity, and levels of neutrophilic chemotactic factors and TLR/MyD88 signal in infected lungs can be corrected by rIL-21 administration. These results revealed that IL-21/IL-21R may aggravate the neutrophil inflammation through regulating TLR/MyD88 signal pathway during chlamydial respiratory infection.

## 1. Introduction

The *Chlamydiaceae* family comprises a group of Gram-negative obligate intracellular pathogens that have a biphasic development cycle including infectious elementary bodies (EBs) and replicative reticulate bodies (RBs) [1–3]. Among the four commonly recognized species of the genus *Chlamydia*: *Chlamydia trachomatis* (*C. trachomatis*), *Chlamydia psittaci* (*C. psittaci*), *Chlamydia pneumoniae* (*C. pneumoniae*), *and Chlamydia pecorum* (*C. pecorum*), the species with the greatest impact on human health is documented to be *C. trachomatis* [2, 4]. Considered to be causative for several human diseases including trachoma, sexually transmitted diseases, and infant pneumonia, *C. trachomatis* usually causes mucosal infections through eye, genital, and respiratory tracts, bring considerable morbidity and socioeconomic burden worldwide [5–7]. In recent years, significant advances have been made in chlamydial respiratory infection immunity researches using *Chlamydia muridarum* (*C. muridarum*), previously known as *C. trachomatis* mouse pneumonitis biovar or MoPn, a rodent-adapted pathogen that causes pneumonitis [8, 9]. However, as the lack of effective vaccine against *C. trachomatis* infection, further study of immunological mechanism in host defense against chlamydia is urgent.

IL-21 is the newest member of the four-*α*-helical bundle type I cytokine family, whose receptor complex containing the common cytokine receptor *γ* chain (*γ*_c_) as functional subunit. As reported, IL-21 is produced primarily by natural killer T (NKT) cells, T follicular helper (Tfh) cells, and Th17 cells [10]. IL-21 receptor (IL-21R) is widely expressed on CD4^+^ and CD8^+^ T cells, B cells, NK cells, dendritic cells, macrophages, and nonimmune cells such as epithelial cells [11, 12]. Based on broad effects on target cells, IL-21/IL-21R was demonstrated to have pleiotropic effects on humoral and cellular immunity as well as additional inflammatory pathways, implying its regulatory properties in infection, autoimmunity, and cancer [12–14]. Recent researches revealed that IL-21/IL-21R plays both proinflammatory and anti-inflammatory roles in infection and inflammation. Among *Mycobacterium tuberculosis* (Mtb) infection, IL-21 caused the lysis of Mtb-infected human monocytes and Mtb growth inhibition through enhancing the IFN-*γ* and anti-microbial peptide production by NK cells [15]. In *methicillin-resistant Staphylococcus aureus* (MRSA) infection, IL-21 could promote neutrophil infiltration and augment granzyme-mediated MRSA clearance [16]. In study of spontaneous experimental autoimmune encephalomyelitis (EAE), IL-21 was observed to promote Th17 cell generation and enhance IL-23R expression on them, thus aggravated the inflammatory reaction and disease progression [17]. During pneumonia virus of mouse (PVM) infection, the numbers of neutrophils, CD4^+^ T cells, CD8^+^ T cells, and *γδ*T cells decreased in the lung of IL-21R deficient mice, resulting in the increased survival rates [18]. In *C. muridarum*-induced chlamydial respiratory infection mouse model, our previous study observed enhanced Th1 and Th17 immune responses, previously proved protective responses against chlamydial infection, in lungs of IL-21R deficient (IL-21R^−/−^) mice. Further results identified increased T-bet/STAT4 (Th1 transcription factors) and STAT3 (Th17 transcription factor) levels in lungs of IL-21R^−/−^ mice following *C. muridarum* infection. These data suggested the regulation of IL-21/IL-21R on CD4^+^ T cell subset responses [3]. However, the latent mechanism and effects of IL-21/IL-21R on innate immune response in *C. muridarum* respiratory infection remain unclear, thus warranting further investigation.

As the first line of host defense against invasive pathogens, neutrophils can be recruited to inflammatory sites and act on elimination of bacteria, fungi, and protozoa infections [19]. These effects were reported to be generated by the combination of chemotaxis, phagocytosis, release of NADPH oxidase-derived reactive oxygen species (ROS) and granular proteins, production of cytokines, and neutrophil extracellular traps (NETs) [20]. However, persistent robust effector functions may also lead to neutrophil-mediated tissue damages at infected sites [21], suggesting the dual role of neutrophils in inflammation. In chlamydial infection, the role of neutrophils in chlamydia-induced pathology has been revealed [22–26]. Using the plasmid-deficient strain of *C. muridarum*, Frazer et al. found that enhanced neutrophil longevity and recruitment contributed to the severity of oviduct pathology [22]. Our previous data have shown that *C. muridarum* infection induced pulmonary damage with significant neutrophil infiltration in susceptible C3H mice [25]. Further, in another study on the role of IL-27/IL-27R in chlamydial respiratory infection, we found that IL-27R knockout mice (WSX-1^−/−^ mice) suffered more severe disease with excessive IL-17-producing CD4^+^ T cells, and there are many more neutrophils and neutrophil-related chemotactic factors in lungs [26], which further suggested the pathologic tissue damage induced by massive neutrophil infiltration in chlamydial respiratory infection.

In this study, we explored the effects of IL-21/IL-21R on neutrophilic inflammation and the potent immune mechanism in mouse model of chlamydial respiratory infection. Our results found the pathological effects of IL-21/IL-21R in *C. muridarum* respiratory infection through inducing excessive neutrophil infiltration, with IL-21R deficient mice exhibited less chemokines and proinflammatory cytokines responses. The decreased expression of TLR/MyD88 pathway mediators in lungs of IL-21R^−/−^ mice further revealed that IL-21/IL-21R might improve the TLR signaling that facilitates chemotactic factor production and consequently leads to neutrophil-mediated pathologic immune response. Thus, our study reveals the modulation and relevant mechanism of IL-21/IL-21R on neutrophilic inflammation, which expand the cognition of host immune mechanism against chlamydial infection.

## 2. Materials and Methods

### 2.1. Animals

Female wild-type mice (WT; C57BL/6) were purchased from the Huafukang Biotechnology (Beijing, China) with the license number of SCXK (Beijing) 2019-0008. IL-21R^−/−^ mice on the same background were obtained with permission as gifts from Dr. Zhinan Yin (Nankai University, China) as previously described [3]. Six to 8-week old mice were randomly divided into different groups (3-4 mice per group) according to different infection time points, and all the mice studied were repeated at least 3 times. All mice were housed at Tianjin Medical University under specific pathogen-free (SPF) conditions with the license number of SYXK (Tianjin) 2019-0004, and all the animal experiments were in accordance with the Animal Ethical and Welfare Committee (AEWC) and approved by the ethical committee of Tianjin Medical University.

### 2.2. Respiratory Infection and Administration of rIL-21

*Chlamydia muridarum* (*C. muridarum*), obtained from Dr. Xi Yang (the University of Manitoba, Canada), was cultured, purified, and enumerated as previously described [27]. For *C. muridarum* respiratory infection animal model, mice were anesthetized via inhalation of isoflurane and then intranasally inoculated with 1 × 10^3^ inclusion forming units (IFUs) of *C. muridarum* in 40 *μ*l sucrose-phosphate-glutamic acid (SPG) buffer. For administration of recombinant murine IL-21 (rIL-21) (PEPROTECH), mice were inoculated intranasally with 0.5 *μ*g rIL-21 in 20 *μ*l PBS at 1 day before infection and days 0, 2, 4, and 6, and the control group was given 20 *μ*l sterile PBS in the same schedule. Mice were monitored daily for body weight changes and euthanized for analysis at designated time points after infection.

### 2.3. Lung Single Cell Preparation and Inflammatory Cell Classification

*C. muridarum*-infected lung lobes were minced with scissors and digested with 2 mg/ml collagenase XI (Sigma-Aldrich) in PRMI-1640 for 55 min in 37°C. Tissue fibers and erythrocytes were successively removed by 35% Percoll (GE Healthcare) and ACK Lysis buffer (Tris-NH_4_Cl). Single cells were washed and resuspended in complete RPMI-1640 medium (RPMI-1640 supplemented with 10% heat-inactivated FBS, 0.05 mmol/L 2-mercaptoethanol, 100 U/ml penicillin, and 0.1 mg/ml Streptomycin) for further analysis. For classification of inflammatory cells, lung single cells were dropped onto the slide and subjected to Wright-Giemsa staining (Baso Diagnostics Inc), and the cell morphology was observed under light microscopy (100x). Neutrophils, monocytes, and lymphocytes were differentially enumerated until the total count reached 200 cells, and the average numbers were calculated from all the sampled fields.

### 2.4. Flow Cytometry

Washed with FACS buffer (PBS with 2% FBS), prepared lung single cells were incubated with unlabelled anti-CD16/CD32 (eBioscience) to block the Fc receptors. After washed and suspended by the FACS buffer, blocked cells were stained with anti-CD45-PerCP (BD Biosciences), anti-CD11b-FITC (BioLegend), anti-Ly-6G-PE (Sungene), anti-CD64-APC (BioLegend), and CD62L-PE-Cy7 (BD Biosciences) for 30 min in the dark at 4°C to indicate the pulmonary neutrophils. Then, the cells were fixed with 2% paraformaldehyde (PFA) in PBS for 30 min at 4°C, and 100 *μ*l FACS buffer was finally added to resuspend the fixed cells. The flow cytometry analysis was conducted on FACS Canto II flow cytometer (BD Biosciences), and acquired data were analyzed using the Flow Jo software version 10 with pulmonary neutrophil subsets being defined as CD45^+^ CD11b^+^ Ly-6G^+^ cell.

### 2.5. Immunofluorescence Staining

The *C. muridarum*-infected lung tissues were fixed with 4% paraformaldehyde for 24 h at 4°C and then dehydrated with sucrose solutions. Dehydrated lung tissues were inflated with OCT (Sakura), and 8 *μ*m lung cryosections were cut. The lung sections were blocked with 10% Goat serum (Sangon Biotech) in 2% bovine serum albumin (BSA; Solarbio) for 1 h at room temperature and incubated with primary antibodies against Ly-6G (Abcam, 1 : 200) that diluted with 1% BSA overnight at 4°C to detect Ly-6G^+^ neutrophils. Next, the slices were washed with PBS and incubated with Alexa Fluor 488 (green)-conjugated secondary antibodies (Abcam, 1 : 500) in 1% BSA for 1 h at room temperature in the dark. Followed by washing five times using PBS, DAPI (Southern Biotech) was used to stain nuclei for 5 minutes and images were captured using fluorescent microscope (20x).

### 2.6. Measurement of MPO Enzymatic Activity

Myeloperoxidase (MPO), highly expressed in neutrophils, was quantified according to the manufacturer's instructions (Jiancheng Bioengineering Institute, Nanjing, China) following *C. muridarum* infection. Briefly, the 5% lung tissue homogenates (5% (w/v) lung tissue in homogenate medium) were prepared by ultrasonic tissue disruptor. After water bath for 15 min at 37°C, the assay buffer and chromogenic agent were added successively, mixed, and started another water bath for 30 min at 37°C. At the final time point, the stop mix was added and incubated for 10 minutes at 60°C to stop the reaction. The absorbance was detected at 460 nm to evaluate the MPO activity in lungs.

### 2.7. RNA Extraction and PCR Analysis

*C. muridarum*-infected lung tissues from mice were homogenized, and Trizol reagent (Invitrogen) was used to extract the total RNA from lung tissues in accordance with the manufacturer's instructions. Reverse transcription of RNA was performed using the cDNA synthesis kit from the TransGen Biotech. The quantitative real-time RCR (qPCR) was performed using the SYBR Green qPCR Mix kit from the SparkJade and proceeded on Light Cycler 96 (Roche), and the expression of target genes is presented as the “fold change” relative to that of control samples (WT mice at 0d p.i.). Primers used were as follows: *β*-actin (as endogenous control) forward: GGCTGTATTCCCCTCCATCG and reverse: CCAGTTGGTAACAATGCCATGT; MMP8 forward: TGGTGATTTCTTGCTAACCCC and reverse: TACACTCCAGACGTGAAAAGC; S100A8 forward: AAATCACCATGCCCTCTACAAG and reverse: CCCACTTTTATCACCATCGCAA; IL-1*β* forward: GAAATGCCACCTTTTGACAGTG and reverse: TGGATGCTCTCATCAGGACAG; IL-6 forward: TCTATACCACTTCACAAGTCGGA and reverse: GAATTGCCATTGCACAACTCTTT; TNF-*α* forward: CTGAACTTCGGGGTGATCGG and reverse: GGCTTGTCACTCGAATTTTGAGA; KC forward: CTGGGATTCACCTCAAGAACATC and reverse: CAGGGTCAAGGCAAGCCTC; MIP-2 forward: GAGCTTGAGTGTGACGCCCCCAGG and reverse: GTTAGCCTTGCCTTTGTTCAGTATC; CXCR2 forward: GCCCTGCCCATCTTAATTCTAC and reverse: ACCCTCAAACGGGATGTATTGT; TLR2 forward: CACCACTGCCCGTAGATGAAG and reverse: AGGGTACAGTCGTCGAACTCT; TLR4 forward: GCCTTTCAGGGAATTAAGCTCC and reverse: GATCAACCGATGGACGTGTAAA; MyD88 forward: TCATGTTCTCCATACCCTTGGT and reverse: AAACTGCGAGTGGGGTCAG; and p65 forward: AGGCTTCTGGGCCTTATGTG and reverse: TGCTTCTCTCGCCAGGAATAC.

### 2.8. Statistical Analysis

Data are presented as mean ± SD, and the statistical analysis was performed with GraphPad Prism version 7. As indicated, two-way ANOVA followed by Bonferroni's multiple comparisons test was used to compare two different groups. Replicates and group sizes were as indicated, and *P* values < 0.05 were regarded as significant (^∗^*P* < 0.05; ^∗∗^*P* < 0.01; ^∗∗∗^*P* < 0.001; ^∗∗∗∗^*P* < 0.0001). The levels of significance are noted on the graphs.

## 3. Results

### 3.1. *C. muridarum*-Infected IL-21R^−/−^ Mice Exhibit Less Pulmonary Neutrophil Infiltration

**Figure 1:**
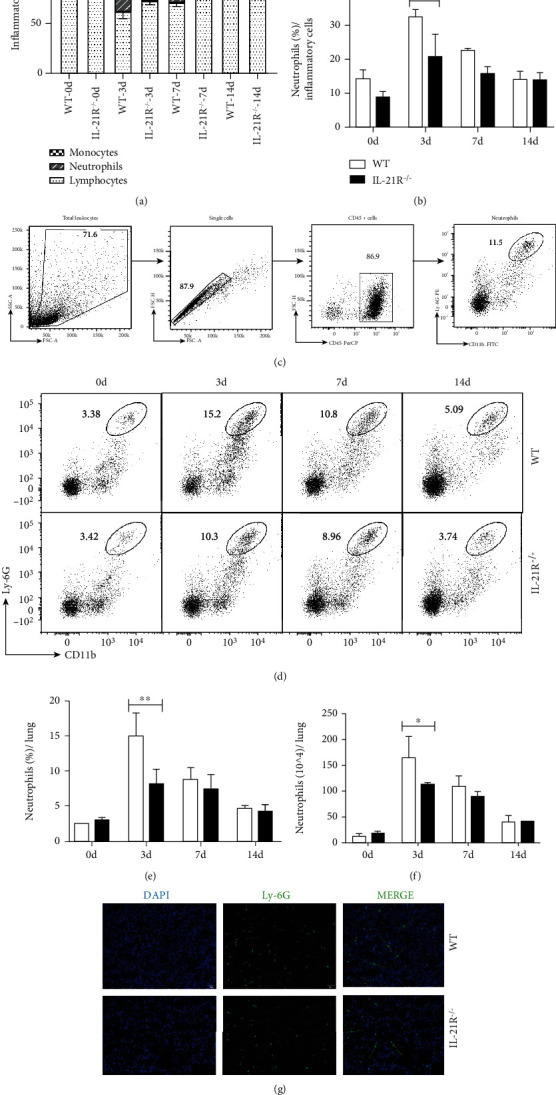
Pulmonary neutrophil infiltrations of IL-21R^−/−^ mice in response to *Chlamydia muridarum* (*C. muridarum*) lung infection. The lung single cells of wild-type (WT) and IL-21 receptor deficient (IL-21R^−/−^) mice were prepared. (a, b) Wright-Giemsa staining of lung single cells was performed, and the percentages of monocytes, neutrophils, and lymphocytes in lungs of WT and IL-21R^−/−^ mice following *C. muridarum* infection were analyzed. (c–f) Flow cytometry performed with pulmonary neutrophils represented as CD45^+^ CD11b^+^ Ly-6G^+^ cells. (c) Representative flow cytometric plots, (d) frequencies, and (e) total numbers of (f) neutrophils in the lungs were shown. (g) Lung cryosections at day 3 p.i. were stained by anti-Ly-6G for neutrophil (green), and nuclei were stained with DAPI (blue), representative immunostaining captured by fluorescence microscopy (20x). Data are represented as means ± SD from *n* = 3–4 per group, representative one of (c–f) five independent experiments or (a, b, g) three independent experiments. Statistical significances of differences are determined by two-way ANOVA followed by Bonferroni's multiple comparisons test. ^∗^*P* < 0.05; ^∗∗^*P* < 0.01.

Our recent study had identified moderate disease of IL-21R^−/−^ mice in the host response to *C. muridarum* infection with enhanced Th1 and Th17 responses (Supplemental Figure 1 and 2) [3]. Resistant IL-21R^−/−^ mice also displayed reduced pulmonary inflammatory pathology with less inflammatory cell infiltration, thus Wright-Giemsa staining of lung cells was performed here and less proportion of pulmonary neutrophils were observed in IL-21R^−/−^ mice than WT mice, especially at day 3 p.i. (Figures 1(a) and 1(b)). This evidence hinted at the neutrophil-mediated pathologic immune response during *C. muridarum* lung infection and the regulatory role of IL-21/IL-21R. As one of the critical innate immune cells, neutrophils have been reported to respond to both viral and bacterial infections, cause tissue damages at infected sites as well [21, 28, 29]. We further investigated the pulmonary neutrophil infiltration of *C. muridarum*-infected WT and IL-21R^−/−^ mice through flow cytometry and immunofluorescence analysis. For flow cytometry, neutrophils were defined as CD11b^+^ Ly-6G^+^ cells (Figure 1(c)) and quantified at representative time points after infection (Figure 1(d)). Following *C. muridarum* infection, neutrophils recruited to the lung and peaked at day 3 p.i., while IL-21R^−/−^ mice exhibited significantly lower levels of neutrophils on both percentage and absolute number in lungs at the same time points (Figures 1(e) and 1(f)). Consistent with that, immunofluorescence analysis of lung sections demonstrated that Ly-6G^+^ cells were differently distributed throughout the lungs between two groups at day 3 p.i. (Figure 1(g)). These findings showed distinct neutrophil infiltration levels in infected lungs of WT and IL-21R^−/−^ mice, suggesting that IL-21/IL-21R plays a stimulative role in excessive neutrophil infiltration in lungs following *C. muridarum* respiratory infection.

### 3.2. The Neutrophil Activity and Biological Function Are Suppressed in Lungs of IL-21R^−/−^ Mice following Infection

**Figure 2:**
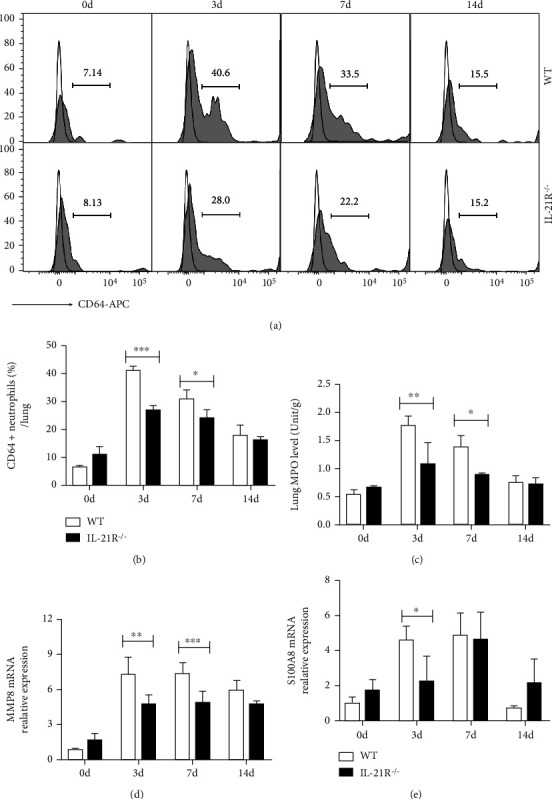
Pulmonary neutrophil activities of IL-21R^−/−^ mice in response to *Chlamydia muridarum* (*C. muridarum*) lung infection. (a, b) The CD64 expressions (shaded histogram) with fluorescence minus one (FMO) control (solid lines) on pulmonary neutrophils from wild-type (WT) and IL-21 receptor deficient (IL-21R^−/−^) mice were analyzed by flow cytometry based on gated neutrophils as described in Figure 1(c). And the percentages of positive cells at indicated times after infection were indicated. (c) The myeloperoxidase (MPO) activity in the lung tissues at days 0, 3, 7, and 14 p.i. (d, e) The mRNA expression of matrix metalloproteinase 8 (MMP8) and S100A8 after *C. muridarum* infection was detected by quantitative real-time PCR (qPCR). Data are represented as means ± SD from *n* = 3–4 per group, representative one of three independent experiments. Statistical significances of differences are determined by two-way ANOVA followed by Bonferroni's multiple comparisons test. ^∗^*P* < 0.05, ^∗∗^*P* < 0.01, and ^∗∗∗^*P* < 0.001.

Previous studies found low expression of CD64 molecule on resting neutrophils; however, it increases once neutrophils become activated [30, 31]. Based on it, we determined the activation status of neutrophils by detecting the surface CD64 expression firstly. Flow cytometry analysis showed that CD64 expressions on neutrophils displayed nearly 6-fold increase at day 3 p.i., while IL-21R^−/−^ mice showed only 3-fold increase compared with uninfected animals (Figures 2(a) and 2(b)). Stored in azurophilic granules of neutrophils, myeloperoxidase (MPO) represented as microbicidal function and activation marker of neutrophils [19]. Our results discovered that *C. muridarum* infection markedly induced the pulmonary neutrophil MPO activity, and as expected, IL-21R^−/−^ mice showed lower lung MPO activity both at day 3 and 7 p.i. (Figure 2(c)). The expression of matrix metalloproteinase 8 (MMP8) and S100A8, two neutrophil-produced proteins involved in modulating inflammatory response [32, 33], showed the same decreases in IL-21R^−/−^ mice compared with the WT group during pulmonary *C. muridarum* infection (Figures 2(d) and 2(e)). Overall, these findings suggested that IL-21/IL-21R is involved in promoting the activity and biological function of pulmonary neutrophils during *C. muridarum* lung infection.

### 3.3. The Reduced Levels of Neutrophil Chemotactic Factors and TLR/MyD88 Signal Pathway Are Related with IL-21R Deficiency following *C. muridarum* Infection

**Figure 3:**
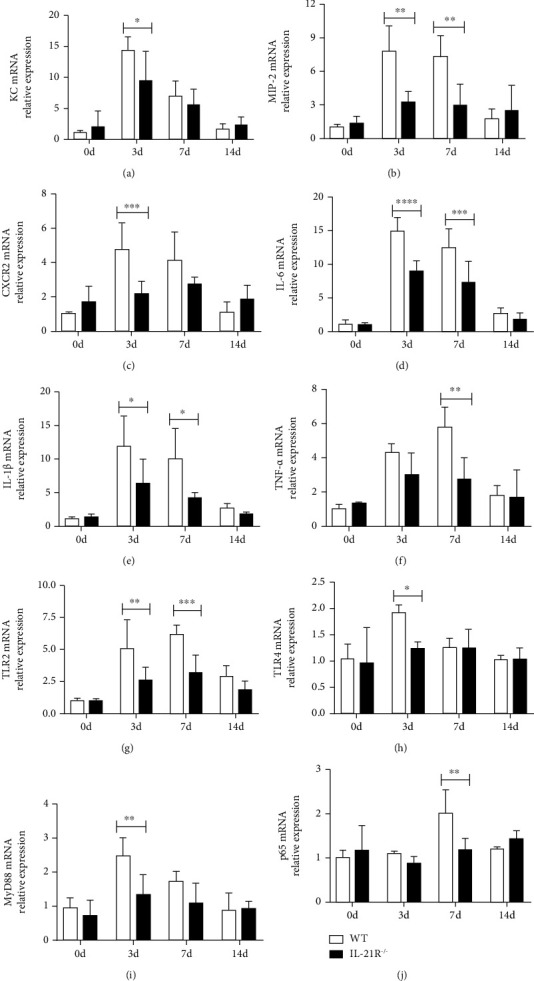
Pulmonary chemokines, cytokines, and TLR/MyD88 signal of IL-21R^−/−^ mice in response to *Chlamydia muridarum* (*C. muridarum*) lung infection. Following *C. muridarum* infection, mice were euthanized at days 0, 3, 7, and 14 and lungs were harvested for mRNA expression analysis. Total RNA was extracted from pulmonary tissues of wild-type (WT) and IL-21 receptor deficient (IL-21R^−/−^) mice, and the mRNA expression of chemotactic factors (a–f) KC, MIP-2, CXCR2, IL-6, IL-1*β*, and TNF-*α* as well as (g–j) TLR/MyD88/NF-*κ*B pathway mediators TLR2, TLR4, MyD88, and p65 was measured by quantitative real-time PCR (qPCR). Data are represented as means ± SD from *n* = 3–4 per group, representative one of three independent experiments. Statistical significances of differences are determined by two-way ANOVA followed by Bonferroni's multiple comparisons test. ^∗^*P* < 0.05, ^∗∗^*P* < 0.01, ^∗∗∗^*P* < 0.001, and ^∗∗∗∗^*P* < 0.0001.

We hypothesized that IL-21/IL-21R alters cell-extrinsic factors that impact neutrophil recruitment, activity, and biological function during *C. muridarum* infection. KC and MIP-2 belong to the glutamic acid-leucine-arginine (ELR)+ CXC chemokine family, which plays key roles in recruitment of neutrophils. Following *C. muridarum* infection, the mRNA expression of KC, MIP-2, and their receptor (CXCR2) in lungs was upregulated in the early phase and remained at high level at day 7 p.i., while their expressions were reduced in IL-21R^−/−^ mice (Figures 3(a)–3(c)). In addition, the pulmonary mRNA expression of proinflammatory cytokines including IL-6, IL-1*β*, and TNF-*α* was lower in lungs of IL-21R^−/−^ mice after infection (Figures 3(d)–3(f)). These data prompted that the decreased neutrophilic inflammation in IL-21R*^−/−^* mice during *C. muridarum* infection may represent as consequence of reduced production of chemotactic factors.

As the link between microbial recognition and the innate immune response initiation, toll-like receptors (TLRs) were widely expressed on a number of immune cells including macrophages, dendritic cells, and neutrophils [34, 35]. And the involvement of TLR2 and TLR4 in the initiation of immune cell activation and immunological response in chlamydial infections were demonstrated in several studies [35–37]. In our study, the qPCR analysis showed lower TLR2, TLR4, and the TLR adaptor molecule MyD88 (mediates all TLR signaling except for TLR3) mRNA expression after *C. muridarum* infection in lungs of IL-21R^−/−^ mice than the control group, indicating that TLR2/4/MyD88-mediated immune response against *C. muridarum* infection might be regulated by in IL-21/IL-21R (Figures 3(g)–3(i)). NF-*κ*B was one of core downstream signal pathways of TLR/MyD88, whose activation was reported to induce the expression of proinflammatory cytokine and chemokine genes to expand inflammatory responses [38]. We discovered different mRNA expression level of NF-*κ*B p65 subunit in two groups of mice after infection, suggesting this signaling pathway could represent as potential target for IL-21/IL-21R regulation on neutrophil response during *C. muridarum* infection, while its role is to be tested further (Figure 3(j)). All these results demonstrated that IL-21/IL-21R could upregulate the chlamydia-induced TLR/MyD88 signal, followed by enhanced chemotactic factor production, thus aggravates the neutrophilic inflammation in lungs during *C. muridarum* infection.

### 3.4. Administration of rIL-21 Aggravates Neutrophil Response in WT Mice after *C. muridarum* Lung Infection

**Figure 4:**
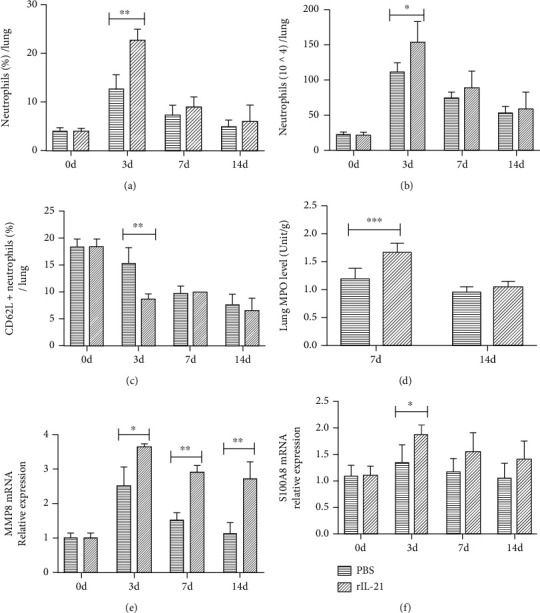
The pulmonary neutrophil level of WT mice after administration of recombinant murine IL-21 (rIL-21) during *Chlamydia muridarum* (*C. muridarum*) lung infection. For rIL-21 treatment, wild-type (WT) mice were inoculated intranasally with 0.5 *μ*g rIL-21 in 20 *μ*l PBS at the day before and days 0, 2, 4, and 6 after *C. muridarum* infection, and the control group was given 20 *μ*l sterile PBS in the same schedule. The (a) percentages and (b) numbers of pulmonary neutrophils were detected by flow cytometry with pulmonary neutrophils represented as CD45^+^ CD11b^+^ Ly-6G^+^ cells. (c) The percentages of CD62L positive cells in pulmonary neutrophils were analyzed by flow cytometry at indicated times after infection. (d) The myeloperoxidase (MPO) activity in the lung tissues after *C. muridarum* infection was shown. (e, f) The mRNA level of matrix metalloproteinase 8 (MMP8) and S100A8 was examined by qPCR. Data are represented as means ± SD from *n* = 3–4 per group, representative one of three independent experiments. Statistical significances of differences are determined by two-way ANOVA followed by Bonferroni's multiple comparisons test. ^∗^*P* < 0.05, ^∗∗^*P* < 0.01, and ^∗∗∗^*P* < 0.001.

To further confirm the contribution of IL-21/IL-21R to infection progression and neutrophil inflammation, recombinant murine IL-21 (rIL-21) or equal volume of PBS was given to WT mice that subsequently challenged with *C. muridarum*. The rIL-21-treated mice showed disease exacerbation with severer lung pathology than control mice following *C. muridarum* respiratory infection as previously described (Supplemental Figure 3) [3]. Higher levels of pulmonary neutrophils were observed in rIL-21-treated mice at day 3 p.i. compared with PBS-treated mice (Figures 4(a) and 4(b)). The same trends of rIL-21-treated mice were also found in neutrophil activation status and biological function, including CD62L (whose downregulation indicates increased neutrophil activity) expression on pulmonary neutrophils, lung MPO activity, and the mRNA expression of neutrophil-produced proteins MMP8 and S100A8 (Figures 4(c)–4(f)). Collectively, these data further suggested the role of IL-21/IL-21R in *C. muridarum* pathology by contribution to excessive neutrophil inflammation in the respiratory tract.

### 3.5. rIL-21-Treated WT Mice Display Higher Levels of Chemotactic Factors and TLR/MyD88 Pathway during *C. muridarum* Lung Infection

**Figure 5:**
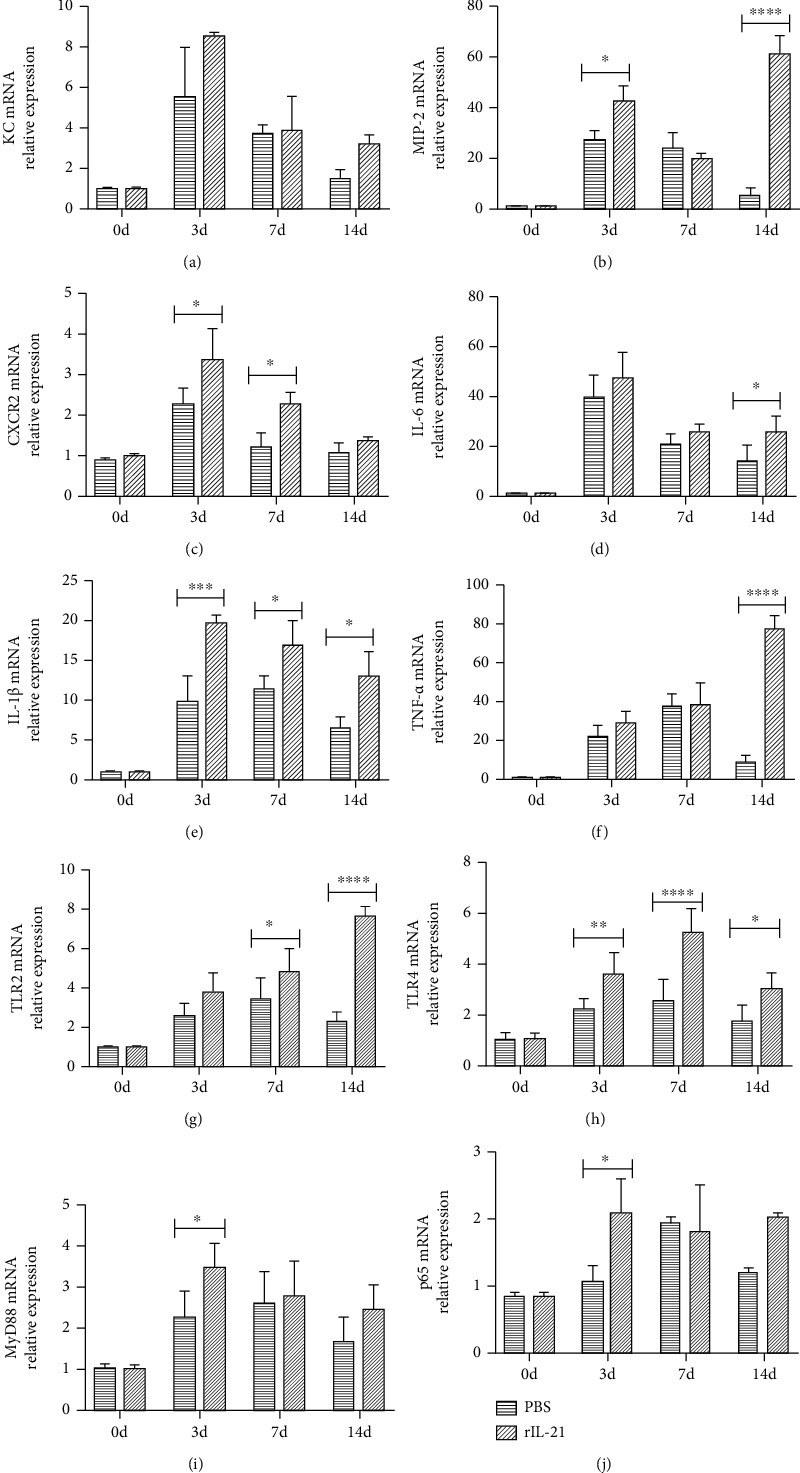
Pulmonary chemokines, cytokines, and TLR/MyD88 signal of recombinant murine IL-21- (rIL-21-) administrated wild-type (WT) mice in response to *Chlamydia muridarum* (*C. muridarum*) lung infection. The rIL-21 and PBS-treated mice were killed at days 0, 3, 7, and 14 after *C. muridarum* infection, and total RNA was extracted from the lung tissues to analyze mRNA expression. The quantitative real-time PCR (qPCR) results of chemotactic factors (a–f) KC, MIP-2, CXCR2, IL-6, IL-1*β*, and TNF-*α* as well as TLR/MyD88 pathway mediators (g–j) TLR2, TLR4, MyD88, and p65 were shown. Data are represented as means ± SD from *n* = 3–4 per group, representative one of three independent experiments. Statistical significances of differences are determined by two-way ANOVA followed by Bonferroni's multiple comparisons test. ^∗^*P* < 0.05, ^∗∗^*P* < 0.01, ^∗∗∗^*P* < 0.001, and ^∗∗∗∗^*P* < 0.0001.

To further verify the cell-extrinsic chemotactic factors that are underlying regulation of IL-21/IL-21R on neutrophilic inflammation during *C. muridarum* infection, mRNA expression of neutrophil-related chemokines, proinflammatory cytokines, and TLRs/MyD88 pathway markers was analyzed in rIL-21 and PBS treated mice. Compared with PBS-treated mice, the rIL-21-treated mice showed upregulated chemokine MIP-2, chemokine receptor CXCR2, and cytokine IL-6, IL-1*β*, and TNF-*α* mRNA expression in lungs after *C. muridarum* infection, which further proved that IL-21/IL-21R might promote neutrophil inflammation through upregulating chemotactic factor expression (Figures 5(a)–5(f)). The TLR2, TLR4, MyD88, and p65 mRNA levels in rIL-21-treated mice were also higher compared with the control group, suggesting that rIL-21 treatment facilitated TLR2/4/MyD88 pathway during *C. muridarum* infection (Figures 5(g)–5(j)). Collectively, these data demonstrated a mechanistic link between IL-21/IL-21R and *C. muridarum*-induced TLR/MyD88 signaling, which aggravate neutrophil-mediated pathological inflammation during *C. muridarum* lung infection.

## 4. Discussion

IL-21/IL-21R was reported to play immunopathological role in *C. muridarum* lung infection in our recent research [3]. Here, we further demonstrated that resistant IL-21R^−/−^ mice exhibited less pulmonary neutrophil infiltration, activity, and biological function compared with the control group, indicating the neutrophil inflammation during *C. muridarum* lung infection and the direct regulatory role of IL-21/IL-21R. PCR analysis proved that in the absence of IL-21/IL-21R signal, the mRNA expression of neutrophil-related chemokines, inflammatory cytokines, and TLR/MyD88 pathway mediators in infected lungs declined, demonstrating their modulation on neutrophil level during *C. muridarum* infection. Complementarily, rIL-21-treated mice showed even graver neutrophilic inflammation, accompanied by increased mRNA expression of chemotactic factors and TLR/MyD88 pathway mediators than PBS-treated mice. To our knowledge, this is the first study to demonstrate that IL-21/IL-21R plays effective promoting role in neutrophil-mediated pathological inflammation through upregulating TLR/MyD88 pathway during chlamydial respiratory infection.

In line with our study, IL-21 was widely reported to mediate pathologic effects in several inflammatory diseases, such as inflammatory bowel disease, rheumatoid arthritis, psoriasis, systemic lupus Erythematosus, pneumonia virus of mice infection, experimental autoimmune encephalomyelitis (EAE), and type 1 diabetes [17, 18, 39]. For example, in the study of type 1 diabetes development, deficiency in IL-21R renders the nonobese diabetic (NOD) mice resistant to onset of type 1 diabetes. Correspondingly, overexpression of IL-21 in pancreatic *β*-cells induced inflammatory cytokines and chemokines and ensued leukocytic infiltration in the islets, resulted in destruction of *β*-cells and spontaneous type 1 diabetes of C57/BL6 mice. These results demonstrated the essential role of IL-21 in diabetes pathogenesis in animal models [40]. In the study of EAE mouse model that simulates cllinical features of Multiple sclerosis (MS), IL-21R deletion caused a defect in IL-17-producing CD4^+^ T cell generation and limited IL-23R expression on Th17 cells, with reduction on the incidence and severity of spontaneous EAE, suggesting that IL-21/IL-21R signaling promotes pathogenic Th17 immune response and development of spontaneous disease [17]. In mouse model of chlamydial infectious disease, this study provides new understanding for the pathogenesis of IL-21/IL-21R through aggravating neutrophilic inflammation.

On research of IL-21R^−/−^ mice following *C. muridarum* infection, our previous study revealed increased IL-17 production [3], which has chemotactic effect on neutrophil via induction of chemokines including CXCL1, CXCL2, CXCL5, and CXCL8 [41, 42]; however, this study proved lower pulmonary neutrophil infiltration. Indeed, the double-bladed sword impact of IL-17 on host defense against *C. muridarum* respiratory infection was reported by our previous studies, Bai et al. firstly demonstrated that moderate IL-17/Th17 response promoted protective type 1 T cell immunity by modulating DC function [43]. However, Zha et al. founded that excessive IL-17/Th17 response caused increased neutrophil inflammation and contributed to severer disease when IL-27R deficient [26]. In this model, the inconsistency between higher IL-17 production and reduced neutrophil recruitment could be explained by the diversity of neutrophil chemotactic factors. Our study investigated common chemokines and classic inflammatory cytokines, which were reported to be involved in neutrophil recruitment and neutrophilic inflammation [44–46]. Decreased mRNA expression of KC (CXCL1), MIP-2 (CXCL2), IL-6, IL-1*β*, and TNF-*α* in lungs of IL-21R^−/−^ mice were consistent with controlled neutrophil infiltration and inflammation. In addition, documented to recruitment neutrophil during inflammation [33], S100A8 mRNA expression was reduced in lungs of IL-21R^−/−^ mice after infection, which also partly accounted for lower neutrophil chemotaxis when IL-21/IL-21R blocked. Further, in addition to chemotactic factors, it is convinced that neutrophil level is modulated organically by granulopoiesis in the bone marrow, release to blood, recruit to the infected or injured tissue, and destruction and clearance [47]. Therefore, further studies on multiple regulation pathway of IL-21/IL-21R on neutrophil number are warranted.

The apoptosis (programmed cell death) of neutrophil, which was reported to largely regulate neutrophil number in inflammatory site, causing expansion or resolution of inflammation [19], was detected in our study to clarify the reason for increased neutrophil response after chlamydial infection. The flow cytometry analysis with Annexin/PI showed apparent neutrophil apoptosis rate in lungs after *C. muridarum* infection, especially at day 3 and 7 p.i., and no difference between the WT and IL-21R^−/−^ mice (Supplemental Figure 4). However, high apoptosis rate did not seem to affect the excessive infiltration of neutrophil in our infection model. As shown in Figure 1, the classification of lung inflammatory cells, flow cytometry, and immunofluorescence analysis all proved remarkable neutrophil infiltration in lungs after *C. muridarum* infection, in line with severe pulmonary pathology. These results indicated that despite higher apoptosis rate of recruited neutrophil, *C. muridarum* respiratory infection induces excessive neutrophil infiltration in infected lung tissues, followed by neutrophilic inflammation. These data also explained why we turn to explore cell-extrinsic factors contributing to neutrophil infiltration. Anyway, more research is needed for exploring the effects of IL-21/IL-21R on neutrophil cell-intrinsic factors, including apoptosis, to improve this study.

Designed to deeper mechanistic exploration, we detected the mRNA expression of NF-*κ*B subunit p65 in lungs of *C. muridarum*-infected mice. As one of core downstream signals of TLR/MyD88, NF-*κ*B has been characterized as important transcriptional factor that regulates proinflammatory genes including cytokines, chemokines, and adhesion molecules to modulate inflammatory responses [38]. In this study, we observed reduced p65 mRNA expression in lungs of IL-21R^−/−^ mice compared with the control group, in line with the decreased chemotactic factor expression. Combined with the facilitation of NF-*κ*B on inflammatory cytokines and chemokines documented in literature, we speculated that IL-21/IL-21R upregulated the chlamydia-induced TLR/MyD88/NF-*κ*B signal, thus induced enhanced chemotactic factors production in lungs during *C. muridarum* infection. However, the participation of NF-*κ*B pathway could not be determined simply by the change of p65 mRNA expression, and further study clarifying NF-*κ*B activation status is necessary. Though we did not define specific signal pathway target implying the regulation of IL-21/IL-21R on TLR/MyD88 signal, our hypothesis provides a direction for future research.

## 5. Conclusions

In conclusion, our study demonstrated that IL-21/IL-21R promotes the neutrophil-mediated pathologic inflammation through upregulating TLR/MyD88 signal pathway during chlamydial respiratory infection. Though future experiments are needed to explore the potential molecular mechanism, our study highlights the necessity of finding the balance of neutrophil responses, which allows for moderate innate immunity against chlamydial respiratory infection while preventing potential pathology. More importantly, these findings promote the in-depth understanding of the pathogenesis of chlamydial infection, which will provide potential immunotherapy targets for chlamydial infectious diseases.

## Data Availability

The data used to support the findings of this study are available from the corresponding author upon request.

## Supplementary Materials

Supplementary Materials Supplemental Figure 1: the mRNA expression of IL-21 and IL-21R in wild-type (WT) mice (C57BL/6) following *Chlamydia muridarum* (*C. muridarum*) respiratory infection. Supplemental Figure 2: host resistance of IL-21R^−/−^ mice against pulmonary infection with *Chlamydia muridarum* (*C. muridarum*). Supplemental Figure 3: the disease progression of WT mice after administration of recombinant murine IL-21 (rIL-21) during *Chlamydia muridarum* (*C. muridarum*) lung infection. Supplemental Figure 4: pulmonary neutrophil apoptosis of WT and IL-21R^−/−^ mice in response to *Chlamydia muridarum* (*C. muridarum*) lung infection.
