# Supplementary material for: IL-21/IL-21R Regulates the Neutrophil-Mediated Pathologic Immune Response during Chlamydial Respiratory Infection
Source: Mediators Inflamm. 2022 Jun 1;2022:4322092. doi: 10.1155/2022/4322092 (PMC9177341; doi:10.1155/2022/4322092)
Supplement: Supplementary Materials — Supplemental Figure 1: the mRNA expression of IL-21 and IL-21R in wild-type (WT) mice (C57BL/6) following Chlamydia muridarum (C. muridarum) respiratory infection. Supplemental Figure 2: host resistance of IL-21R−/− mice against pulmonary infection with Chlamydia muridarum (C. muridarum). Supplemental Figure 3: the disease progression of WT mice after administration of recombinant murine IL-21 (rIL-21) during Chlamydia muridarum (C. muridarum) lung infection. Supplemental Figure 4: pulmonary neutrophil apoptosis of WT and IL-21R−/− mice in response to Chlamydia muridarum (C. muridarum) lung infection. [file 4322092.f1.docx]

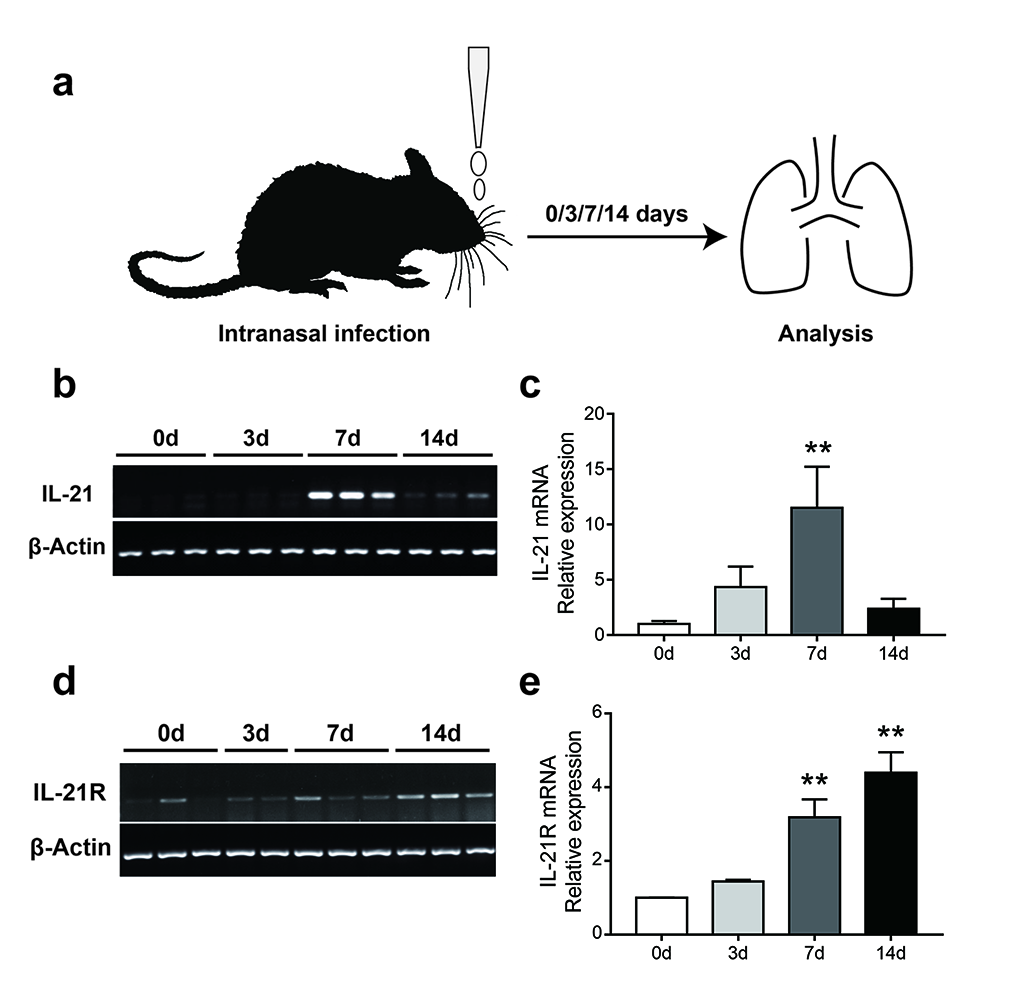


**Supplemental Figure 1:** **The mRNA expression of IL-21 and IL-21R in wild-type (WT) mice (C57BL/6) following** ***Chlamydia muridarum (C. muridarum)* respiratory infection.** (**a**) Animal model of *C. muridarum* respiratory infection was induced by intranasally inoculating with 1×10^3^ inclusion forming units (IFUs) *C. muridarum*. Mice were euthanized at days 0, 3, 7 and 14 post-infection (p.i.) and lungs were harvested for following analysis. Total RNA was extracted from pulmonary tissues and the mRNA expressions of IL-21 and IL-21R were measured by Reverse Transcriptase PCR (RT-RCR) (**b** and **d**) and Real-time PCR (qPCR) respectively (**c** and **e**). Data are represented as means ± SD from n=3-4 per group, representative of three independent experiments. Statistical significances of differences are determined by one-way ANOVA with Dunnett's multiple comparisons test. **P＜0.01.


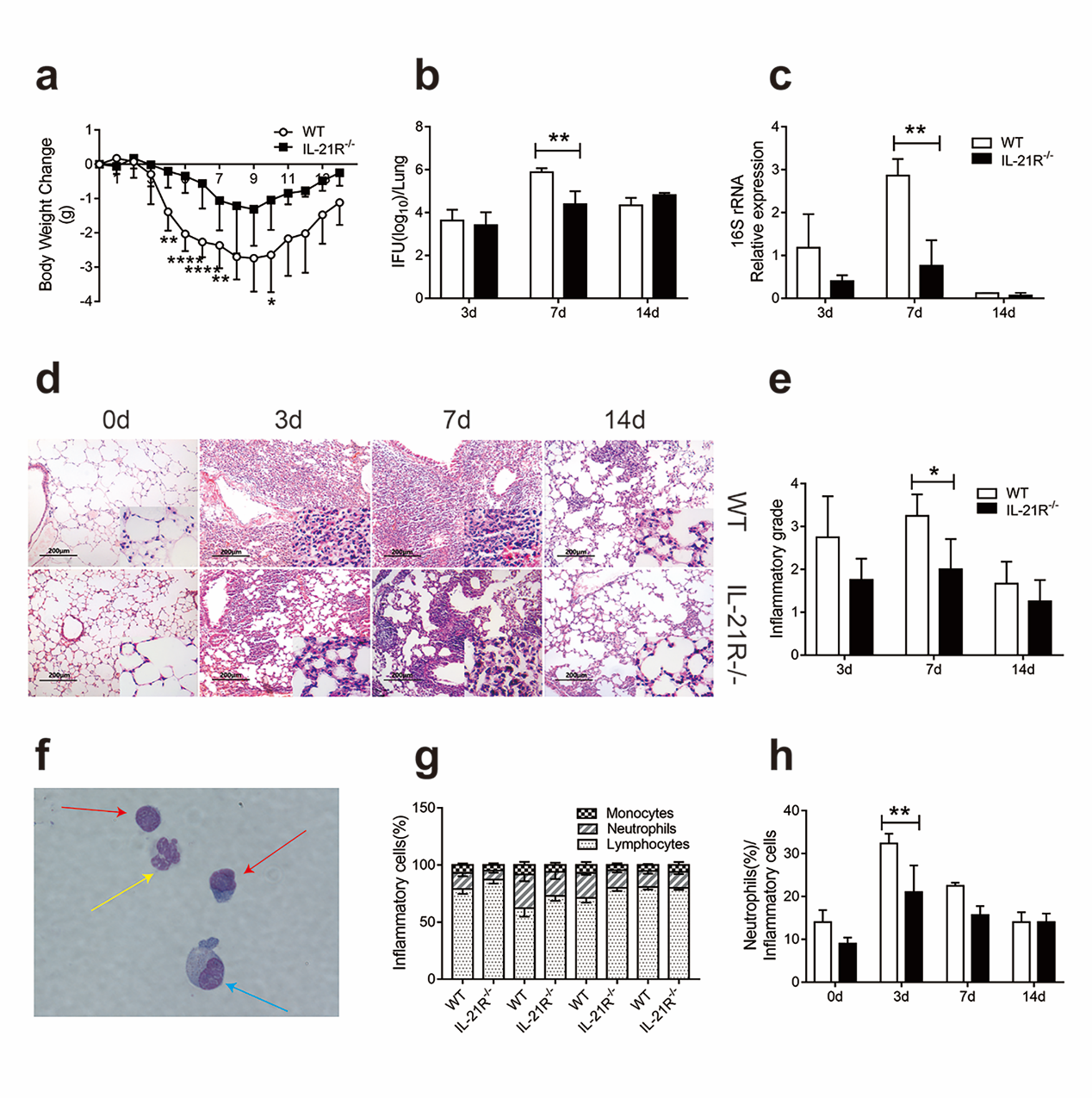


**Supplemental Figure 2: Host resistance of IL-21R^-/-^ mice against pulmonary infection with *Chlamydia muridarum (C. muridarum)*.** (**a**) Following *C. muridarum* infection, the body weights of infected wild-type (WT) and IL-21 receptor deficient (IL-21R^-/-^) mice were monitored daily. (**b**) Pulmonary *Chlamydia* inclusion forming units (IFUs) and (**c**) *C. muridarum* 16S rRNA level by Real-time PCR (qPCR) were evaluated as pulmonary chlamydial loads. (**d**) Lung sections were stained by H&E, the pathological changes were captured under light microscopy [10X and 20X] and the inflammatory grades were scored with the semi-quantitative pathological scoring method (**e**). Data are represented as means ± SD from n=3-4 per group, representative of five independent experiments. Statistical significances of differences are determined by two-way ANOVA followed by Bonferroni's multiple comparisons test. *P＜0.05, **P＜0.01, ****P＜0.0001.


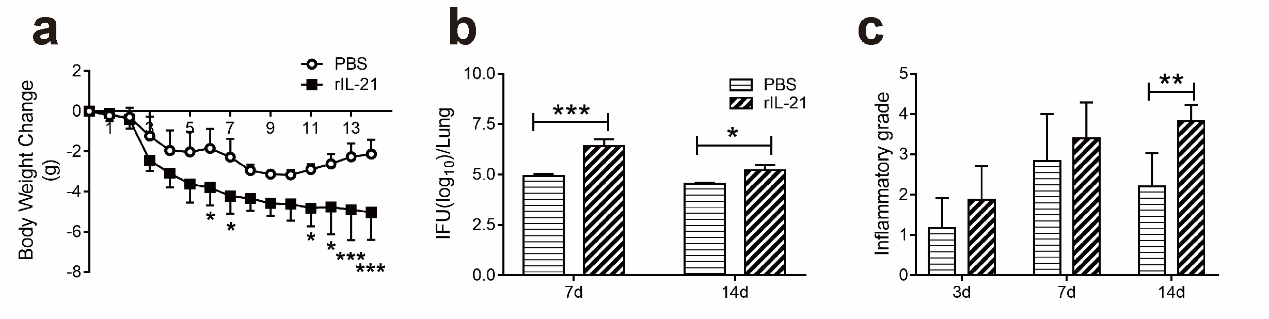


**Supplemental Figure 3: The disease progression of WT mice after administration of recombinant murine IL-21 (rIL-21) during *Chlamydia muridarum (C. muridarum)* lung infection.** For rIL-21 treatment, wild-type (WT) mice were inoculated intranasally with 0.5ug rIL-21 in 20 ul PBS at the day before and days 0, 2, 4 and 6 after *C. muridarum* infection, the control group given 20ul sterile PBS in the same schedule. (**a**) The body weight changes were monitored daily. At days 0, 3, 7 and 14 post infection, rIL-21 and PBS treated mice were killed. (**b**) The lung homogenate was prepared for determining chlamydia inclusion forming units (IFUs). (**c**) Semi-quantitative pathological scoring was used for H&E-stained lung sections with a blinded manner to the experiment and sample ID. Data are represented as means ± SD from n=3-4 per group, representative of three independent experiments. Statistical significances of differences are determined by two-way ANOVA followed by Bonferroni's multiple comparisons test. *P＜0.05, **P＜0.01, ***P＜0.001.


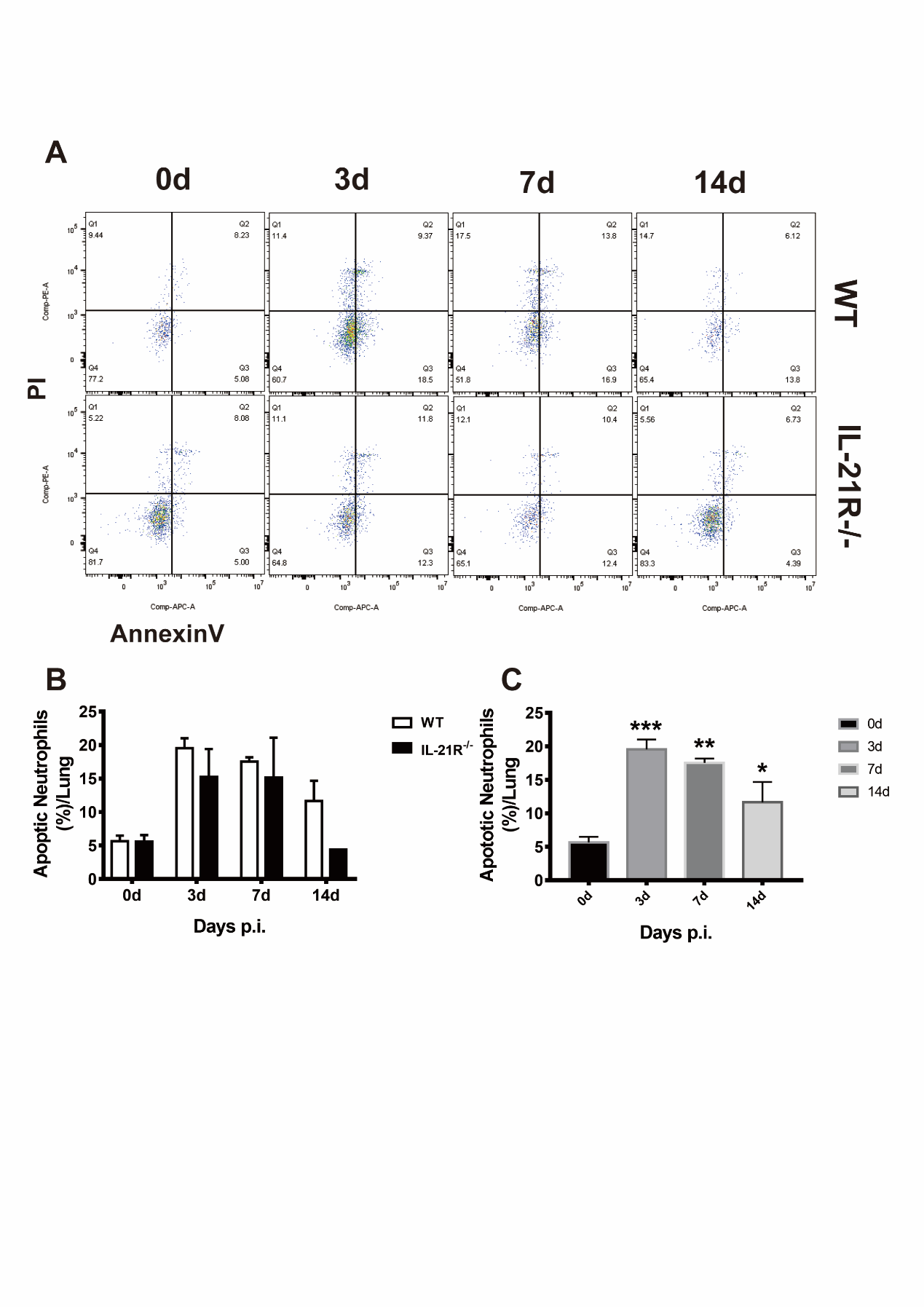


**Supplemental Figure 4: Pulmonary neutrophil apoptosis of WT and IL-21R^-/-^ mice in response to *Chlamydia muridarum (C. muridarum)* lung infection.** The apoptotic neutrophil (Q3: PI^lo^ AnnexinV^hi^) in lungs from wild-type (WT) and IL-21 receptor deficient (IL-21R^-/-^) mice were analyzed by flow cytometry based on gated neutrophils (CD45^+^ CD11b^+^ Ly-6G^+^) as described in Fig. 3A. Representative flow cytometric plots (**A**), frequencies in two group of mice (**B**) and in WT mice (**C**) of pulmonary neutrophils were showed. Data are represented as means ± SD from n=3-4 per group. Statistical significances of differences are determined by two-way ANOVA followed by Bonferroni's multiple comparisons test (**B**) or one-way ANOVA with Dunnett's multiple comparisons test (**C**). *P＜0.05, **P＜0.01, ***P＜0.001.
